# Indigenous Parents’ Perspectives of Factors That Facilitate or Impede Engagement in Internet-Based Parenting Support Programs: Interpretive Description Study

**DOI:** 10.2196/64994

**Published:** 2024-11-22

**Authors:** Michelle L Butt, Ysabella Jayne Willett, Vicky Miller, Brenda Jacobs, Era Mae Ferron, Amy L Wright

**Affiliations:** 1 School of Nursing McMaster University Hamilton, ON Canada; 2 Lawrence Bloomberg Faculty of Nursing University of Toronto Toronto, ON Canada; 3 Hamilton Regional Indian Centre Hamilton, ON Canada

**Keywords:** child, parenting, qualitative, Indigenous health, support programs

## Abstract

**Background:**

Parenting support programs enhance parents’ health and their child’s development. The COVID-19 pandemic necessitated the delivery of these programs over the internet. After the pandemic, internet-based programs are still preferred by some.

**Objective:**

We aimed to understand Indigenous parents’ experiences engaging in internet-based parenting support programs; thus, an interpretive description study was conducted.

**Methods:**

A total of 20 Indigenous (female, male, and Two-Spirit) parents of children aged <5 years participated in semistructured interviews; data underwent collaborative thematic analysis with Indigenous community partners informed by the Two-Eyed Seeing framework and ethical space.

**Results:**

Parents’ experiences were classified into five themes: (1) Purpose: Program Delivery and Content, (2) Belonging: Building Relationships and Connections, (3) Hope: Cultural Connection, (4) Meaning: New or Improved Parenting Skills and Mental Wellness, and (5) Recommendations for Organizations.

**Conclusions:**

The study findings can inform internet-based parenting program delivery to enhance engagement for Indigenous families.

## Introduction

### Background

Parenting programs provide critical support to individuals parenting a child. Research indicates that these programs have numerous positive outcomes for parent and child health and wellness, such as improved parent mental health and positive child development outcomes [1,2]. Parenting programs are especially important for parents of young children (aged <5 years) as this stage in life is a critical time for learning and child development [3]. Early childhood experiences impact brain development, and healthy relationships with adults are pivotal to that development [3]. Problems or delays not addressed in early childhood could potentially have long-term implications for health and wellness [4].

Parenting programs typically target specific populations, such as parents of children aged <5 years, parents of children with unique needs (eg, autism spectrum disorder), or parents belonging to groups or communities who are marginalized by mainstream society. Indigenous-specific parenting programs incorporate cultural teachings and traditions, including the presence of elders, ceremonies, and crafts [5]. These programs are congruent with Indigenous parenting approaches that draw from traditional roles and a community-oriented approach with family kinship models and nonnuclear families and are distinct from Western parenting approaches [6].

Parenting programs in Ontario, Canada, include the generic and Indigenous-specific Healthy Babies Healthy Children, Head Start, and EarlyON programs, which offer education and activities by licensed early childhood educators to promote healthy parenting and early childhood development [7-10]. Aboriginal Health Centres and Indigenous-led community centers such as the Indigenous Friendship Centres in Ontario also offer a wide array of programs and services, including parenting programs with a culturally grounded approach [11,12]. Indigenous parents can choose to access any of these programs according to their needs and availability.

Parenting programs can be offered in various formats: in person; internet based (self-guided); or facilitated and supported by a service provider over the internet, which may include early childhood educators or those otherwise trained to provide parent and child programs. During the peak of the COVID-19 pandemic, many parenting programs were exclusively offered over the internet and have continued with that mode of delivery. The efficacy of internet-based parenting programs on child and parent health has been previously well documented in the literature [1,2,13]. A meta-analysis of the effectiveness of internet-based parenting programs, for example, found that the programs increase positive parenting, parent confidence, parent satisfaction, and positive child behaviors and were effective in reducing negative parent-child interactions, parent stress, child behavior problems, and child anxiety [1].

Despite the benefits of parenting programs, parent attendance and engagement are not consistent across all parenting programs. The opportunity to meet other parents and exchange ideas, the ability to learn new skills, and trust in the person delivering the program [14] facilitate engagement in parenting programs. However, barriers such as limited resources; feeling judged or discriminated against; inadequate program funding; programs that are not culturally relevant; and program-specific factors such as delivery, content, and support impact attendance and participation in these programs for some families [15,16]. Typically, attendance and engagement by parents who need the services the most is low [15].

In view of the positive impacts of parenting programs on the parent and child, parental attendance and engagement in programs is important. With an increase in internet-based parenting programs since the COVID-19 pandemic, an understanding of how to effectively engage parents in parenting programs over the internet is needed. This need was also identified by the Hamilton Regional Indian Centre (HRIC), a nonprofit organization sitting on the traditional territories of the Haudenosaunee and Mississauga Nations that provides culturally relevant Indigenous programs and services, including programs aimed at supporting urban-residing Indigenous parents of young children and their families. These programs address topics such as prenatal guidance, parent-infant bonding, building communication skills, emotional regulation, and nutritional education, to name a few. Service providers follow a curriculum provided by the HRIC and adjust content to meet their clients’ needs, including integrating teachings that align with the specific cultures of the program’s attendees. During the COVID-19 pandemic, parenting programs at the HRIC switched from in-person to internet-based delivery, facilitated and supported by service providers in real time with varying success. Service providers at the HRIC have shared their challenges with internet-based program delivery, including initiating and maintaining parental engagement.

Given the importance of Indigenous parents receiving culturally relevant parenting support, especially for parents of young children, it is important to members of leadership and staff at the HRIC to understand the experiences of Indigenous parents who attend internet-based parenting programs—what is helpful for their engagement and what poses challenges. However, no studies were located that explored the experiences of Indigenous parents engaging in parenting programs over the internet. Thus, examining the perspectives of parents who have attended internet-based programs in a study will help identify the barriers to and facilitators of engagement in internet-based parenting programs, contribute to the existing gap in the literature concerning the experiences of Indigenous parents, and provide the HRIC with information to optimize delivery and improve parental engagement in their internet-based parenting programs.

### The Indigenous Wellness Framework as an Organizing Structure

Fostering the wellness of parents is a key objective of Indigenous parenting support programs; thus, the Indigenous Wellness Framework [17] was selected for structuring this study and organizing the study findings described in this paper as it reflects the HRIC’s holistic understanding of health and wellness. The Indigenous Wellness Framework was developed through a partnership between the Assembly of First Nations and the First Nations and Inuit Health Branch of Health Canada, along with Indigenous mental health leaders, as a shared vision for mental wellness and services for First Nations [17]. The framework was designed to strengthen mental wellness programs and support but has also been used to provide guidance for communities who wish to adapt or optimize their mental wellness programs and services to address their own priorities. At the core of the Indigenous Wellness Framework are 4 *directions* or main wellness outcomes: *purpose*, *belonging*, *hope*, and *meaning*. Within the framework, *purpose* is described as the sense of purpose that people gain through their education, employment, caregiving activities, or cultural ways of being and doing. *Belonging* is the sense of connectedness with their families, community, and culture. *Hope* is described as hope for the future and that of their families, grounded in a “sense of identity, unique values and a belief in spirit” [17]. Finally, *meaning* is an understanding of how their lives and that of their families is part of Creation and a rich history.

### Study Purpose and Research Questions

The purpose of this study was to understand Indigenous parents’ experiences engaging in internet-based parenting support programs. Specifically, the research questions were as follows: (1) What do parents identify as factors that facilitate or impede engagement in internet-based parenting support programs? and (2) What strategies do parents recommend for enhancing the experience of engaging in internet-based parenting programs?

For this study, internet-based parenting support programs were defined as programs that provide parents with information or support for their role as parents. These internet-based programs had to be offered via a platform whereby content was delivered and supported by an individual from an organization in real time. As the HRIC provides programs and services to clients representing many First Nations, Inuit, and Métis communities and cultures, the term *Indigenous* in this paper is used to refer to parents or parenting programs that reflect more than one cultural group.

## Methods

### Steering Committee and Positionality of the Researchers

A steering committee was established to oversee the development of the research plan and conduct of the research in collaboration with the HRIC. The steering committee comprised authors VM, BJ, MLB, and ALW; members VM and BJ from the HRIC led the committee. All steering committee members had previously collaborated on research, most notably members VM, BJ, and ALW, who have a long-standing relationship of >10 years working collaboratively on research projects aimed at understanding the needs of Indigenous parents to better service the community though programs. In addition to the steering committee oversight, research and administrative support for the study was provided by authors YJW and EMF.

When conducting research with Indigenous Peoples, a relational and community-led approach to research is ethically imperative and morally necessary [18,19]. As such, it is important that we situate ourselves by providing the positionality of the research team. First, the first author, MLB, is a non-Indigenous nurse researcher with clinical expertise in neonatal nursing and healthy parenting and a parent of 2 children. She has worked alongside the HRIC on projects for 4 years. YJW is a Mi’kmaw and settler woman from the Acadia First Nation, Squlj Clan, living and working in the Sɨpekne’katik district within the traditional territory of Mi’kma’ki of what is now known as Nova Scotia, Canada. She has an academic background in public health and strives to uplift the voices of Indigenous Peoples in her work. VM and BJ are First Nations from Six Nations of the Grand River in Ontario, Canada. VM is of the Cayuga Nation, Turtle Clan, and BJ is from the Mohawk Nation, Bear Clan, and a Knowledge Holder. Both act as managers at the HRIC overseeing portfolios consisting of cultural safety training for local non-Indigenous organizations, programming and staffing at the HRIC, and child protection advocacy. They are both mothers and have close relationships with clients at the HRIC. EMF is a non-Indigenous nurse and mother of 3. Finally, ALW is a non-Indigenous nurse researcher and clinician with European settler ancestry and lives with her husband and children in the traditional territories of the Haudenosaunee and Mississauga First Nations in Southern Ontario, Canada. She strives to use her unearned privilege as a White scholar and academic to support the research needs of the Indigenous communities and organizations with whom she collaborates in a community-led approach to research. Together, the team worked collaboratively, deferring to those with Indigenous lived experience and integrating the perspectives of other staff members and parents at the HRIC throughout the project.

### Guiding Principles for the Research

The Two-Eyed Seeing framework [20] and the principles of the ethical space of engagement [21] guided the research. First, the Two-Eyed Seeing framework guided the steering committee in valuing seeing the world with one eye grounded in an Indigenous worldview and the other eye with a Western worldview [20]. In particular, the Two-Eyed Seeing framework guided our collaborative approach to the study. The focus of this research was identified through cooperative dialogue with service providers from the HRIC and Indigenous parents in Hamilton, Ontario, to discuss community strengths and challenges related to the availability and delivery of parenting programs. This study, including the development of the research questions and data collection procedures, was designed in collaboration with First Nations staff from the HRIC to ensure that they were culturally appropriate and safe. The steering committee oversaw the project, including assisting with recruitment, data analysis, and conceptualization of modalities of knowledge dissemination outputs that best suit the needs of the community.

Second, the principles of the ethical space offered guidance on cooperative dialogue and engagement between 2 diverse groups with differing worldviews—First Nations community members, including a Knowledge Holder (BJ), and non-Indigenous researchers originating from and trained within a Westernized worldview [21]. The ethical space was a safe space for engagement for both groups, with a valuing of the concept of equality and a recognition and respect for the diversity created by philosophical and cultural differences [21]. Throughout this collaboration, the research team deferred to First Nations partners and their understandings of the local context, cultures, traditions, and protocols. Their understanding was especially important during analysis as this informed the findings.

### Research Design

Interpretive description methodology was selected to address the study aims to understand what facilitated and impeded parents’ ability to engage in parenting programs that were offered in an internet-based format and what strategies parents identified to improve parental engagement in internet-based parenting programs [22]. Interpretive description is a qualitative research approach that transcends the description of a phenomenon [22]. The approach not only allows for an understanding of the characteristics, themes, and patterns within subjective perceptions of a phenomenon but provides an interpretive account that can guide and inform practice [22]. Interpretive description emphasizes the integration of expert knowledge, including the perspectives of those participants who have lived experience of the phenomenon. As such, our use of this methodology facilitated the cocreation of knowledge with HRIC service providers, who have a deep understanding of the contextual forces impacting parents’ experiences of internet-based programs. Along with parents’ experiences, this collective understanding can inform change at the program delivery level, promoting more effective and engaging internet-based program delivery for Indigenous parents and young children.

### Setting and Sample

A purposive sample of 20 parents who had used an internet-based parenting support program was recruited. Parents met the following inclusion criteria: (1) self-identification as Indigenous or being members of a family who self-identified as Indigenous, (2) engagement in an internet-based offering of a program related to parenting in southern Ontario, and (3) having been expecting or parenting a child aged <5 years when they participated in the parenting program. Parents who had only engaged with in-person parenting programs were excluded.

Purposeful, maximum variation sampling allowed for the selection of a diverse group of parents [23] who had used internet-based parenting programs. Specifically, we sought parents of varying ages, support (partner involved or not), socioeconomic statuses, previous parenting experiences, previous use (or not) of in-person, non–internet-based parenting programs, and experiences of parenting a child with or without health or developmental challenges. To complement the purposive sampling, snowball sampling was also used to increase the sample size and offer access to potential participants that otherwise may not have been reached. A sample size of 15 to 20 was considered a reasonable target to capture the diversity of parents who attended the programs. The sample size was ultimately determined when information power and conceptual redundancy were achieved [24].

### Recruitment

Parents were recruited using two primary methods: (1) via parenting program staff at the HRIC and (2) through social media. A research flyer identifying the study purpose, eligibility criteria, and contact information for the research coordinator was distributed by HRIC staff via email to their program attendees, or printed copies were physically shared with parents attending parenting programs, as well as posting it on the information board at the center. The flyer was also made available on social media platforms, including one research team member’s professional Twitter (subsequently rebranded X) account and on HRIC social media platforms. Participants were encouraged to share the flyer with other parents they knew had experience participating in internet-based parenting programs.

### Data Collection

Parents providing informed consent participated in individual, in-depth, one-on-one semistructured interviews via Zoom (Zoom Video Communications) or telephone at a mutually agreeable time. Author YJW, a research team member with experience in qualitative study interviewing, conducted the interviews. Before commencement of the interview, YJW first prioritized establishing rapport with the participant, taking a relational approach to research and as a way to facilitate reciprocity through promoting a positive experience for the participant [18,19]. YJW then reviewed the consent form and reminded the study participant that they may choose not to answer some or all the questions. A semistructured interview guide was used to elicit parents’ perspectives on their engagement in internet-based parenting programs. The interview guide was developed based on the study purpose and literature on internet-based parenting support. The interview guide was pilot-tested with 2 First Nations community members and modified accordingly to make certain that the questions adequately explored the topic of focus and were clear and culturally appropriate. The interviewer also verbally administered a short demographic questionnaire at the beginning of the interview. The demographic questionnaire included questions such as the age of the parent, self-identified gender, educational level completed, number of children they were parenting at home, relationship with a partner, number of people living in the home, and cultural background. Each interview was 30 to 80 minutes in length depending on how expressive the participant was and was video or audio recorded.

### Data Management

All digital recordings of the participant interviews were transcribed using an automated transcription service. Transcripts were reviewed by a research assistant for accuracy, and identifying information such as names or places was removed. The NVivo software (version 12.0; QSR International) [25], a computer-assisted qualitative data management program, was used to organize, analyze, and store the data during analysis.

### Data Analysis

In keeping with the interpretive description research design, data collection and analysis occurred concurrently in an iterative process [26]. Data collection continued until conceptual redundancy and information power occurred [24]. Inductive thematic data analysis was used guided by the Two-Eyed Seeing framework [20]. Coding strategies as described by Saldana [27] were used to conduct several iterations of coding until final themes were identified. The themes were the patterns of meaning identified and derived from the data. Demographic data from the semi-structured interviews were entered into SPSS (version 26; IBM Corp) and summarized using descriptive statistics.

Using a Two-Eyed Seeing approach, data analysis was a collaborative process with all members of the steering committee, and authors VM and BJ guided the analysis to ensure that local Indigenous ways of knowing and service providers’ expertise were incorporated and accounted for in the results. Non-Indigenous researchers deferred to Indigenous ways of knowing throughout the analysis.

### Rigor

Strategies were used in the research to promote the rigor or trustworthiness of the study findings, including approaches to promote credibility, transferability, dependability, and confirmability [28]. Credibility or the truth value of the study findings was promoted through investigator triangulation, whereby more than one member of the research team conducted the data analysis and the findings were validated with the entire steering committee to confirm the interpretation of the findings. Transferability was promoted through documentation of a thick description of the research context and study participants. Strategies to enhance dependability included memos to describe the process of analyzing and interpreting the data as part of an audit trail. Reflexivity (journaling) of the team members helped enhance the confirmability of the data collection and interpretation of the study findings [28].

### Ethical Considerations

Research ethics approval was obtained from the University of Toronto Research Ethics Board (#43372), and the study was reviewed by the Hamilton Integrated Research Ethics Board (#15047). Researchers adhered to the principles of the Tri-Council Policy Statement 2 [29] and the 4 Rs of research (respect, relevance, reciprocity, and responsibility) [18]. All eligible participants were informed of their right to end the interview at any time or refuse to answer any question they were uncomfortable answering. All participants gave written and oral consent to the research coordinator or interviewer before beginning the interview. Throughout the study, measures were instituted to maintain the confidentiality of the data and the privacy of the study participants. Participants were assigned a code number, and several privacy and safety features of Zoom were enabled during internet-based interviews. The First Nations principles of ownership, control, access, and possession (OCAP) [30] governed the ownership, protection, and storage of the research data, which will be held locally at the Six Nations Polytechnic knowledge institute for access and use by community members to meet community-identified needs at study completion. In addition, findings from the community gathering have been translated into graphic form and made available in paper and electronic formats, including on a public-facing website [31], for the HRIC to distribute to their clientele and use to further inform their programs and services. All study participants were provided with a CAD $50 (US $35.80) gift card as a thank you for their participation.

## Results

### Participants

A total of 20 parents participated in individual interviews; 19 (95%) were Indigenous (First Nations: 15/20, 75%; Métis: 4/20, 20%), and 1 (5%) was non-Indigenous but their household family members were Indigenous. Those who identified as First Nations represented the Cree, Ojibwe (including the Anishinaabe and Chippewa Peoples), Oji-cree, Mohawk, Oneida, Onondaga, Seneca, Algonquin, and Mississauga Nations. Most parents (16/20, 80%) identified as women, whereas the remainder identified as men (2/20, 10%) or Two-Spirit (2/10, 10%). Parents had a mean age of 34.7 (SD 10.33; range 20-67) years. More than half (11/20, 55%) of the participants had completed college, had some university education, or had completed a university undergraduate or graduate degree; approximately one-third (6/20, 30%) had some college education, whereas the highest level of education of a small portion of the parents (3/20, 15%) was the completion of high school. Participants were primarily residents of 1 of 2 large cities in southern Ontario, Canada—Hamilton (10/20, 50%) or Toronto (6/20, 30%)—with other parents residing in the surrounding Greater Toronto Area or Niagara region. None of the participants lived in on-reserve communities. The household size of participants ranged from 2 to 7 individuals. Approximately one-third (7/20, 35%) of the parent participants were caring for only 1 child, whereas the remainder had 2 (6/20, 30%), 3 (5/20, 25%), or 5 (2/20, 10%) children.

Participants shared experiences of using a range of internet-based programs, including Indigenous-specific programs and non-Indigenous programs. Indigenous-specific programs included government-run early childhood programs, known as EarlyON in Ontario, Canada, and programs run by Indigenous-led organizations, including Aboriginal Health Centres and Indigenous Friendship Centres. Non-Indigenous programs encompassed those run by the Boys and Girls Clubs of Canada, non-Indigenous EarlyON programs, prenatal programs, and a young parent–focused program. Most participants (13/20, 65%) drew on experiences from at least 2 parenting programs, whereas 35% (7/20) had used only 1 parenting program.

### Thematic Summary

#### Overview

Study participants identified numerous factors that facilitated or impeded their engagement in internet-based parenting support programs. These factors encompassed many facets of the program and its delivery—from the point in time when parents learned that the program was being offered to the effect of program engagement on them as individuals and parents. Parents also provided many suggestions for organizations to enhance programs and encourage other parents to engage in the internet-based parenting support programs more actively.

The steering committee, as noted previously in this paper, selected core elements from the Indigenous Wellness Framework [17] as a structure for sharing the study findings. Specifically, the 4 main outcomes of the framework are the structure under which the themes are presented. Collectively, the findings of the analysis of the individual interviews are classified into four main themes and organized under the Indigenous Wellness Framework: (1) *Program Delivery and Content (Purpose)*, (2) *Building Relationships and Connections (Belonging)*, (3) *Cultural Connection (Hope)*, and (4) *New or Improved Parenting Skills and Mental Wellness (Meaning)*. A fifth and final theme, *Recommendations for Organizations*, was also identified in addition to those presented under the Indigenous Wellness Framework. Each theme and its respective subthemes are presented in the following sections, including exemplars from study participants.

#### Purpose: Program Delivery and Content

Effective parenting program delivery that purposefully met parents’ informational needs through providing their desired program content was a major theme among study participants. Parents identified their need to be aware of programs being offered, the importance of accessible delivery, and the program content they valued.

#### Awareness of the Program

Before parenting program delivery, organizations advertised their program offering to parents. Parents in this study noted the importance of being aware of programs that would be of benefit to them. Most parents in the study did not voice concerns about *how* they became aware of available programs, but rather, a few parents found it difficult to learn about *what* programs were being offered. One parent, for example, noted the difficulty they had in finding a parenting program:

...you have to really do your researching and find them kind. It seems...they were all given through certain providers and stuff like that throughout the community. So unless it’s like, how do you say, marketed type thing through them, it’s very hard to find them.

Many parents learned about the programs offered through social media. Despite the efficiency and reach of that medium, other parents felt that sharing through physical flyers would also have benefits, as noted by a participant:

...I dunno, maybe there’s lots of people out there who it’s just easier when you’re walking, you see a flyer, it’s like, oh, I didn’t know. And then you just take it and go, or you see it and you take a photo and just, yeah.

#### Accessible Delivery

Accessible program delivery was important for parents and facilitated their engagement in programs. Parents identified two areas that were needed to optimize the accessibility of the parenting program for them: (1) equipment plus internet connectivity and (2) skills and support to use technology.

### Equipment Plus Internet Connectivity

Access to a laptop or other electronic device was essential for a parent to engage in internet-based programs. Participants emphasized the importance of having a device and that the lack thereof impeded engagement with the program. In addition, parents needed internet access to be able to participate in internet-based parenting programs, and access to this service was a potential barrier perceived by some parents. This participant compared her experience to that of her family member’s, who did not have access to a laptop to participate in the internet-based program:

I guess if you don’t have a laptop or a computer, that could be an issue. Cause I think I remember telling my cousin about it because she had kids young too. She has kids my younger son’s age. And I was like, oh, why don’t you do this? You could sign up...but she’s like, oh, my laptop, the kid’s actually broken her laptop. So she was like, I don’t know. I’m like, oh. She said, can I go on my phone and do it? I’m like, maybe if your camera’s working, I guess. But then I think she did. She complained about it saying like, oh, on the phone, it’s so small, the screen. And was, that was an issue. So I think back to your question, I think it’s definitely no issue signing up and accessing it if you have a laptop that’s working, that’s good connection. But if you don’t, then yeah, there could be some barriers there because your phone wouldn’t be the best and it wouldn’t really keep the kids’ attention cause it’s so small, you can’t really stationary it so that they can just sit down or something. They have to hold it and their kids are always moving around. So it’s just kind of chaotic.

Another parent noted the following:

It would be nice to ensure everybody has this kind of access. I don’t know how feasible that is. I know there were parents struggling when school went online and scrambling to get their children tablets and not everybody has internet. And that was a big struggle...I don’t think we’re doing anybody any good by not having that accessibility.

A lack of the appropriate equipment or electronic connectivity meant that some participants had to travel outside their home to obtain the needed equipment and resources to participate, as described by one parent:

And I know for a fact there are people that go to the library just to be able to have access to the basics that you and I probably take for granted. And maybe people are going to the library and engaging in programs that way, but it sucks that you have to, in the middle of minus 30-degree weather, have to trek down to your local library to be able to access that.

### Skills and Support to Use Technology

The ability to use technology to connect with the internet-based parenting program was a new skill for some participants and created an immense learning curve. One parent verbalized how this learning curve was a stressor:

The one thing that really got in the way of it being able to be really helpful is the virtualness, it’s hard to deal with, it’s just learning how to use the different programs and stuff online seeing if my phone can actually run certain programs. That was definitely something that came in the way of me being able to completely throw myself in.

This limited experience or challenges with using technology was felt by some participants as a barrier to actively engaging with the program, as one parent noted:

It really all just had to do with the technology. I think it was just because with...in-person groups and everything, it’s really different cause you, you’re have something in your hand and you’re focusing on something else and try to read this and trying to read that. You’re really more focused when you’re in person. But then online it was really just for lack of a better explanation, it was just all the technology and trying to get used to one program and then go to a different program to do something else. And then it was just very, very confusing.

Despite the challenges experienced by parents due to limited skills in the use of technology, parents were pleased to obtain support from individuals conducting or supporting the delivery of the parenting program. The ability to connect with a program facilitator or staff member via telephone to troubleshoot problems was felt by parents to be important in facilitating their participation in programs.

#### Content Valued by Parents

### Overview

The parenting support programs provided education for parents on caring for and parenting their children. Parents valued several specific content areas of the parenting support programs that prompted their engagement. These were (1) prenatal care and infant health and development and (2) parenting strategies.

### Prenatal Care and Infant Health and Development

Parents felt that it was important to learn about what to expect during labor and understand how to prepare for this event. This information was valued not only by expectant mothers but also by their partners who would be providing support. Parents valued the opportunity to learn about infant health, including breastfeeding, nutrition, and infant safety related to sleep and water. Parents also felt it important to have a cardiopulmonary resuscitation (CPR) training and certification course. Parents valued the prenatal and infant education thoroughly and enjoyed learning this information as it helped prepare them for their new role as parents.

Another content area that parents valued was learning about infant and child development and developmental milestones. Parents appreciated knowing what to keep an eye out for as their children grew to ensure that they were healthy and on track for their age.

### Parenting Strategies

Parents valued parenting support program content that provided various strategies related to parenting effectively. This included strategies to promote engagement with their child, how to address frustrations encountered during parenting, and coping mechanisms to use. For example, one parent described their learning related to strategies to promote engagement with their child:

I know this is about parent engagement, so just from a child and parenting standpoint...I’ve learned more about just how to better engage with your children and appreciate the time you have and to take time.

Parents also expressed the positive impact of strategies learned through parenting:

They warned us about crying and how frustrating it can get and just that if they’re okay and they just keep crying to take a minute to yourself, go take 30 seconds a minute, take a few breaths, come back. That has really helped me work on my patience with my daughter.

#### Belonging: Building Relationships and Connections

The second major theme noted in the data was that the relationships developed with other parents and the parenting program facilitator aided participants’ engagement in the programs. In general, it was important for parents to develop connections and build relationships, particularly during the pandemic as the social distancing measures dramatically changed how parents received services and connected with other parents or supports before, during, and after the birth of their child.

#### Other Parents

Parents wanted to develop relationships with other parents in the programs. New mothers or parents most notably felt that developing these relationships was extremely critical. These new parents were particularly postnatally isolated during the pandemic with limited opportunity to interact with other parents.

Although parents sought interpersonal relationships with other parents in the program, this might not be feasible with or desired by all parents attending a support program. However, it was still deemed important by parenting program participants to generally know the other parents in the program. Even if parents did not seek friendship with other parents, they felt that they had to get to know them as they were sharing personal information about themselves and their families during the program.

#### Program Facilitators

Parents sought and felt that it was important to have a relationship with parenting program facilitators and other workers supporting the parenting program. One parent described why they felt that this relationship was so important:

I think it was important to have that relationship because when you’re talking about child development and parenting, that’s a spectrum and there is so much unknown to that. It’s good to have people in your corner that can support you and have that, that knowledge.

The need for a good interpersonal relationship with program staff and facilitators was largely attributed to the desire to be close with those surrounding and supporting their families—those who knew personal, intimate information about them. Parents described that workers with whom they had a good relationship were almost like friends—people they could go to in a time of need and not feel fear or judgment, as one parent explained:

The relationship that I think we built was a solid one...So she was kind...all the way around. So it didn’t just become a therapeutic relationship, it was also kind of a confidant...Somebody you could turn to if you needed help or advice.

#### A Sense of Belonging and Shared Experience

Developing a sense of belonging to a community and having a sense of shared experiences was a subtheme that was repeatedly discussed by almost all parents who participated in the study. Parents valued the opportunity to have a sense of community during a time when in-person interactions and social connections were limited. They also appreciated the ability to share individual experiences with others, to know that they were not alone in some of the situations they encountered and the subsequent emotions that ensued during that time. One parent spoke about how participating in the parenting support programs made them feel:

I think it felt really good, especially with the pandemic of not having anyone and not just being secluded to your family. So I feel like it gave you a sense of community, a sense of belonging, just kind of like, okay, something to look forward to, something to pencil into your days.

Another parent expressed how the ability to have a sense of community was especially important when others whom they typically relied on were not available due to the restrictions of the pandemic:

Also just still being able to connect with people in a time where we weren’t able to, and especially with our families even...It was the same way with the Indigenous perspective and lines. Traditionally, you have your community there, you have your aunties, your uncles, your brothers and sisters, and we didn’t have that in that time. So it was good to connect with people and still be able to talk and ask questions in a judgment free space, which is something that you would get from your family or could expect, hopefully expect from your family. I mean...mentally it made me feel better in ways too, just to be able to socialize and laugh and joke with people about things and to share feelings. And over time you built a relationship.

This value of a sense of community and connectedness with other parents was also echoed by another parent:

So I think that that sense of community and not feeling alone is probably the most important thing. You can take all the parenting classes, they can give you all the advice that’s important, but you need to give parents an opportunity to connect and talk just [to] them, whether that’s a focus group where you get together and you talk about different topics every week, or a book club where you read different, it can be parenting books, it can be self-help books, it can be whatever. And then you get together and you talk about it. As much as those may not be formally parent related, I do believe that a happy parent is a good parent. And to have that, I feel like you do need to feel like you’re not alone.

This sense of belonging was extremely important as parents noted that they were more likely to share and participate more if they felt connected with other parents and workers.

#### Hope: Cultural Connection

Parents felt that participating in parenting programs provided them with an important opportunity to connect more deeply with their culture and traditions; having this cultural connection was the third major theme in the study data. Parents wanted cultural elements such as traditional teachings, crafts, or cooking included in the programs they accessed. They placed a higher value on programs that integrated cultural components than programs that did not:

I think this is fairly obvious is the cultural connections that you’re able to attain through online. I’m a busy lady and I can’t necessarily commit to evenings a week...to take language courses. So, I’d see another benefit is just being able to connect culturally on a different platform...It’s another connection...I think especially with our agencies...that cultural component. And even in our food, the food that they try and bring that culturally relevancy to their programs, and I really appreciate that. So, I think there’s a good balance of just fun and engaging and cultural. I think there’s a big balance there.

Parents felt that the parenting support programs offered information that served as a reminder of culture that they could share with their children:

There’s things in my childhood that I’m grateful for culturally that I want my child to have that same, to be that fortunate, to have that privilege. And those are words that shouldn’t really be used when you were talking about culture, but when we’re talking specific to First Nations, to Indigenous people, it’s scarce now. So that’s something I want to have instilled in our child. And I just reflect back on different sessions and it’s like I said, those reminders. So it, it’s weird because I think there’s not maybe a week that goes by that I don’t think about these programs in some ways, something that they’ve offered.

The cultural elements of the programs also helped address gaps that parents felt they had in their own knowledge that they could teach their children:

A lot of things I’ve learned that my mother couldn’t teach me...I’m teaching my children that well; I’d like to say my partner is fully native and he doesn’t know anything about his background really. So it has to be my job. So that’s why I need [Program Name]. This is why I need [Parenting Program Name]. This is why I need the HRIC to show them because I don’t know myself.

The cultural elements desired among study participants varied. Participants wanted programs that included crafts and art, such as medicine bags, beading, ribbon skirts, moccasins, and dreamcatchers. Participants wanted to learn more about traditional foods and recipes, such as bannock and Three Sisters soup. Parents wanted programs to be land based if possible and have opportunities to learn their Indigenous languages. Parents desired opportunities to learn more about traditional teaching and ceremonies, such as the Seven Grandfather Teaching, moon ceremonies, powwows, and drumming. They also wanted to learn more about traditional medicines. Learning about how to raise children through a cultural lens was also deemed important.

#### Meaning: New or Improved Parenting Skills and Mental Wellness

##### Overview

Parenting support programs helped create meaning in parents’ lives through the development of new parenting skills or enhancing their current repertoire of skills. The programs also helped create meaning through supporting parents’ mental wellness. This was the fourth and final major theme in the study data.

##### New or Improved Parenting Skills

Nearly half of the parents interviewed reported that engaging in internet-based parenting programs improved their parenting skills and strategies. This included response strategies that were more appropriate than the ones they had either previously used or grown up with. Some parents also described how their newfound skills and abilities—such as cooking—inspired them to be more involved with their children and come from a place of love and patience rather than aggression:

The whole point of that was to learn different methods that my child’s going through to learn different techniques, to not be the type of parent that my father was. So, I’ve definitely sat back and watched my son go out into the world and come back and check in and then go back out into the world and come back and check in. Just like we learned all about in

Parenting Program Name

These new skills being learned had a positive impact on individuals’ ability to parent:

I feel that I’m a better parent now learning new things, how to not go at it aggressively, but to go at it at different approaches.

##### Mental Wellness

During the pandemic, parents also reported that they enrolled and participated in internet-based programs when components of health and wellness were included or focused on self-growth. As mental health was a prominent issue during the pandemic, parents appreciated and valued programs that included components of self-care, mental wellness, and strategies for coping with stress. The positive impact of focusing on well-being in the programs was explained by one parent:

It was a really good space to just express...to talk about experiences in the past and really go into...there were some sessions there where we...focused on wellbeing and so we’d sort of look back and talk about things that happened in the past and why, how they maybe have been carried over the years and impact things that, how we think what we do, the way we do things in the present. So, it was really nice to have those opportunities for inner reflection and building that self-awareness and how that can impact your relationships with others, including your kids.

#### Recommendations for Organizations

##### Overview

Throughout the study, parent participants discussed program elements that facilitated their engagement. Recommendations for organizations to help promote parental engagement in parenting support programs included improved accessibility, delivery that is interactive and engaging and has inclusive support, and incorporation of family-oriented activities. In addition, parents wanted to ensure that food and mental wellness were incorporated as components of the programs. Each of these recommended parenting program elements are discussed in more detail in the sections that follow.

##### Accessibility

###### Overview

Parenting programs had to be accessible for parents to engage with them and reap their benefits. Parents recommended more scheduling options for programs, alternate ways to access programs, and strategies to optimize accessibility of the programs. Sign-up for programs had to be straightforward, and the internet-based parenting program platform had to be uncomplicated and easy to navigate.

###### Scheduling

Challenges were experienced by parents in relation to the scheduling of programs, with few options available that fit parents’ schedules. This challenge was also applicable to the times offered for parents to pick up or for the program team to deliver material or food kits to be used in the program. Thus, parents recommended that more program timeslots be available. However, parents recognized that funding and resources might limit the ability of staff to provide additional program offerings and, therefore, suggested archived recordings (where appropriate) as an alternate way for parents to access the program and relevant information; this would address scheduling challenges.

Parents provided other recommendations to facilitate program accessibility and scheduling. This included posting monthly calendars of all programs available rather than distributing individual flyers for each program to make it easier for parents to plan and choose the program that would work best for their needs, interests, and availability. Another suggestion by parents was for program managers and facilitators to administer a poll to all parents enrolled (or interested) in a program to determine their availability. A unique time that works (mostly) for everyone could then be offered rather than having a prescheduled offering of the program.

###### Sign-Up, Log-In, and Navigation

Parents were less likely to participate or continue in a parenting support program if the sign-up and log-in processes were difficult or overcomplicated. The sign-up for programs had to be straightforward to reduce the potential for parents to choose not to participate due to challenges experienced in enrolling in the program. For example, navigating websites to sign up for programs was sometimes difficult, and some parents preferred the option of calling in to register for programs. Parents recommended that the internet-based parenting program platform have an uncomplicated log-in process and that the program needed to be easy to navigate on the internet. Keeping track of log-in emails and passwords or using several websites, apps, or platforms during the program was difficult for parents. Several parents felt that they were not “tech savvy” and, therefore, a lot of the programs offered were particularly difficult to navigate. These factors should be considered by organizations when setting up future programs to make sign-up, log-in, and navigation as simple and streamlined as possible.

##### Delivery

###### Overview

Parents mostly preferred in-person over internet-based programs for a number of reasons, including improved supports, services, and activities; fostering deeper and more meaningful connections with other parents and workers; better instruction; and the ability for their children to play or interact with other children to develop their social skills. However, parents recognized the value of internet-based programs and thoroughly appreciated the accommodation provided during the pandemic. Parents provided recommendations for enhancing internet-based program delivery, including interactive and engaging programs, inclusive support, and activities.

###### Interactive and Engaging Programs

Parents repeatedly expressed the importance of having programs (and facilitators) that were interactive and engaging. This included not only programs specifically for parents or those who were expecting a child but also family-oriented programs that involved children. Activities that parents viewed as *interactive* or *engaging* included tactile activities, drumming and singing, arts and crafts (with children), cooking, and activities that incorporated opportunities to talk to other program attendees.

###### Inclusive Supports

Parents desired supports and services that were more inclusive. Some explained that they would like programs to be inclusive of a wider children’s age range (ie, beyond 12 months). Other parents wanted to see more Two-Spirit and lesbian, gay, bisexual, transgender, and queer support for children; specific activities for children with attention-deficit/hyperactivity disorder and autism spectrum disorder; and multigenerational programs that included elders and other family member roles rather than only the parent and child. Several parents identified the need to increase the number of current supports and programs available to teenage and young moms, as well as individuals who are expecting to be parents for the first time or are new mothers.

###### Activities

Parents sought programs that were activity based and family oriented so that they could spend quality time with their children. Programs that were hands-on and interactive, such as drumming and singing circles or cooking classes, were often chosen to maintain engagement and excitement and avoid feelings of boredom or repetition. Parents appreciated it when there was an educational component (ie, traditions, healthy recipes, and infant care) included in these activities.

##### Program Components

Parents wanted food to be a part of the program components and content included that addressed health and wellness.

###### Food

Parents described the value of having food incorporated into internet-based programs. Some programs included a weekly food delivery kit as a cooking class to provide recipes and offer a fun activity to do with children. Others taught how to make traditional teas and medicines, such as elderberry juice. Although some organizations offered food delivery services during the pandemic to support food security, the inclusion of food in the programs was viewed by parents as an important consideration for future internet-based programs.

###### Health and Wellness

Parents expressed the need to include more content on self-care (particularly after delivery and in the early stages of parenthood) and mental wellness tips in parenting support programs. Parents wanted to see the incorporation of emotional support and sharing groups into programs—as a means of support and social interaction. Parents also identified healthy eating and the importance of proper nutrition in supporting the body, mind, and spirit as an area for inclusion in parenting programs.

## Discussion

### Principal Findings

This study provides important insights into parents’ perspectives on what facilitates or impedes their participation in Indigenous parenting support programs that are delivered over the internet. While some of the findings of this study echo those in the literature on mainstream internet-based programs, many are novel as this is the first study to consider the unique perspectives of *Indigenous* parents using internet-based parenting programs. In particular, parents’ priorities to use internet-based programs to build relationships and a sense of community with other new parents; the importance of cultural connections to families; and specific ways in which programs can be more inclusive of families impacted by attention-deficit/hyperactivity disorder and autism spectrum disorder and those who are multigenerational, as well as Two-Spirit and lesbian, gay, bisexual, transgender, and queer parents, are important additions to the literature.

One of the major findings of this study was the importance of program delivery, which included accessibility to programs. The need to have the appropriate equipment and internet access was critical. This is not unlike the challenges that many, such as the educational sector, experienced when they had to pivot to internet-based delivery in the early weeks and months of the COVID-19 pandemic; many families did not have computers at home for their children’s schooling [32]. Organizations delivering programs need to consider the need for these technology resources when planning programs and have the resources necessary to provide to parents through a strategy, such as a loan program for devices with internet access. Given the limited resources that many service sector organizations have, working with public libraries to facilitate clients’ access to computers and the internet would be another potential strategy to facilitate internet access. However, this strategy may create another barrier if parents planning to participate are geographically not located close to a library or have limited resources available for transportation to a public library. For example, many Indigenous communities in Canada face high internet costs and slow, unreliable speeds or a lack of internet access entirely [33]. Parents may need to modify screen use to accommodate slow speeds and resort to less ideal formats, such as direct messaging rather than video calls [33]. Organizations need to consider their local context to design strategies to improve access to their programs.

Another component revealed in this study related to accessibility was parents’ steep learning curve in setting up and using the technology or the platform for delivery of the program. Given that parents felt that support from staff via telephone helped them navigate these technological challenges, organizations should consider dedicating a staff person to online support when initially commencing an internet-based program. This will help facilitate program access for parents who have limited experience with technology.

Content that parents valued was another study finding that was identified by parents as facilitating engagement. Not surprisingly, expectant parents valued learning about prenatal care, whereas parents in general valued learning about infant health and child development, in particular elements such as child safety and sleep. These are important areas that are typically included in many programs to support expectant or new parents, such as a public health offering of *Healthy Babies Healthy Children* [7]. However, the difference in this study is that parents emphasized the importance of Indigenous culture and an Indigenous lens when presenting content. Thus, an Indigenous parenting support program would provide this valued lens when delivering content, which parents would not receive in other generic parenting programs.

Parents also valued learning about Indigenous culture and saw this knowledge as a facilitating factor to their program engagement. This is not surprising considering the devastating impacts that colonialism has had on Indigenous Peoples and the resulting loss of cultures, language, and identity [34]. Indigenous parents living in urban areas may not have as strong a connection with their culture as those living on a reserve and may be seeking to reconnect with and learn about their history, culture, and traditional ways [5,35-37]. A study exploring family engagement and well-being in the Aboriginal Head Start programs in British Columbia, Canada, suggests that engagement was most active when families established relationships with elders, facilitating the sharing of Indigenous ways of knowing [5]. This sharing of knowledge and teachings that celebrates culture created a sense of belonging for families and helped programs be culturally inclusive [5]. Access to culturally inclusive parenting programs can increase family wellness and provide support and healing from the harms of colonization [16]. These studies emphasize the need to continue offering programs that integrate culture throughout their educational content. In addition, incorporating activities that create the opportunity for parents to learn about or revisit cultural teachings is extremely important. However, to avoid a pan-Indigenous approach to programs that results in cultural erosion, service providers should reach out to elders or Knowledge Holders from their clients’ home communities and Nations and incorporate information and activities that reflect their unique cultures into their programs.

Relationships with other parents and program facilitators were important to participant parents. Throughout the COVID-19 pandemic, interpersonal relationships were limited to household contacts and some internet-based connections with family and friends for those who had technology. This restriction on interpersonal interactions created a stressor for many individuals and the need for a connection with others. This connection is important, most notably in times when support is more likely to be needed, such as during the transition to parenthood or even when engaging in the day-to-day challenges that many individuals encounter as they parent their children [38]. Thus, the delivery of programs over the internet during the COVID-19 pandemic was very important. It provided individuals with an opportunity to build relationships that became key sources of support when encountering the challenges of parenting or enduring the isolation that the pandemic created. Given this, organizations should consider having opportunities within their programs for individuals to build relationships with other parents attending the program and with program facilitators and staff. Having contact information for the facilitator, if feasible, would also be helpful so that parents can have contact with an individual to support them or refer them to resources as needed.

The parenting support programs provided many benefits or positive outcomes for attendees. It is not surprising that the programs created a sense of belonging and improved mental wellness, especially due to the limited interpersonal interaction posed by the pandemic. Given that mental wellness is important and some parents—such as those in the early postpartum period—are at risk of depression [39], these programs provide a key source of support to help optimize positive mental health.

Despite the overall positive experience and beneficial outcomes that parents had from engaging in parenting support programs, parents did offer recommendations for enhancing programs. Parents verbalized that they wanted interactive, engaging programs that were family oriented. They felt that incorporating food into programs was important. In addition, health and wellness should be considered when developing future programs. These recommendations are important for organizations to consider as they engage in budgeting and planning for future program delivery. Eliciting the help of a few parents in an advisory capacity might be beneficial for exploring how to implement these recommendations cost-effectively and in a way that is responsive to parents’ needs.

### Strengths and Limitations

This interpretive description study incorporated rigorous design elements that contribute to the strength of the research and the credibility of the study findings. Our community-engaged approach ensured that the study met the needs of the local community, including helping improve programs at the HRIC, and that it was guided by leaders and parents at the HRIC. Our purposive sampling approach allowed us to understand the diverse experiences of Indigenous parents, although most participants identified as First Nations. Inuit and Métis parents’ experiences were not adequately captured in this study, and therefore, their experiences with internet-based programs may differ from those in the findings presented here. In addition, we recognize the diversity of First Nations Peoples, and as such, the experiences of the parents in this study may not reflect those of other Nations and communities. Similarly, the experiences of men were not well represented, with only 10% (2/20) of the participants identifying as men. This represents a gap in understanding that should be further explored. Next, we were able to achieve information power and redundancy of concepts [24] and themes during our concurrent approach to data collection and analysis. We engaged in triangulation throughout our collaborative approach to data analysis, which included all members of the steering committee and other First Nations parents. Finally, parents provided their experiences of participating in several different programs that offer support to Indigenous parents, which was a study strength. However, how many programs parents participated in and their frequency of use was not consistently discussed with all participants and could have helped provide further insight into the context of parents’ experiences with specific internet-based parenting support programs.

### Conclusions

Collectively, parents engaging in internet-based, available, Indigenous parenting support programs had positive experiences. The programs facilitated parents’ engagement, providing them with opportunities to develop new parenting skills, develop relationships with other parents and program staff, create a sense of belonging, and deepen their connection with their culture. Organizations offering internet-based Indigenous parenting support programs should consider integrating these recommendations into their programs to better engage parents and children in services.

## Abbreviations

CPR: cardiopulmonary resuscitation
HRIC: Hamilton Regional Indian Centre
OCAP: ownership, control, access, and possession
